# Prevalence of meeting aerobic physical activity and resistance training guidelines in advanced prostate cancer: a descriptive study of racial and regional disparities in the IRONMAN registry

**DOI:** 10.1007/s00520-026-11251-3

**Published:** 2026-09-22

**Authors:** Aleksander Solberg, May Grydeland, Tormod S. Nilsen, Anne H. Storås, Ian D. Davis, Natalie Greaves, Simon Anderson, Andre P. Fay, Kim N. Chi, Deborah Enting, Raymond McDermott, Simone Badal, Charles G. Waihenya, Ademola Popoola, John M. Lazarus, Joaquin Mateo, Anders Bjartell, Aurelius Omlin, Folakemi Odedina, Camille Ragin, Lorelei A. Mucci, Kjell M. Russnes, the IRONMAN investigators

**Affiliations:** 1 Department of Physical Performance, The Norwegian School of Sport Sciences, Oslo, Norway; 2 Department of Sport Science and Physical Education, University of Agder, Kristiansand, Norway; 3 Department of Oncology, Oslo University Hospital, Oslo, Norway; 4 Eastern Health Clinical School, Monash University, Melbourne, Australia; 5 Cancer Services, Eastern Health, Melbourne, Australia; 6 George Alleyne Chronic Disease Research Centre, The University of the West Indies, Cave Hill Campus, Bridgetown, Barbados; 7 African Caribbean Cancer Consortium, Philadelphia, USA; 8 PUCRS School of Medicine, Hospital Nora Teixeira, Santa Casa de Porto Alegre, Porto Alegre, Brazil; 9 BC Cancer, Vancouver Prostate Centre, Department of Medicine, University of British Columbia, Vancouver, Canada; 10 Department of Oncology, Guy’s and St Thomas’ NHS Foundation Trust, London, UK; 11 St. Vincent’s University Hospital & Cancer Trials, Dublin, Ireland; 12 Department of Basic Medical Sciences, The University of the West Indies, Kingston, Jamaica; 13 Department of Surgery, University of Nairobi, Nairobi, Kenya; 14 Department of Urology, University of Ilorin Teaching Hospital, Ilorin, Nigeria; 15 Prostate Cancer Transatlantic Consortium, Ilorin, Nigeria; 16 Department of Urology, Groote Schuur Hospital, University of Cape Town, Cape Town, South Africa; 17 Vall d’Hebron Institute of Oncology, Barcelona, Spain; 18 Department of Translational Medicine (Urology), Skåne University Hospital, Lund University, Lund, Sweden; 19 Onkozentrum Zurich, University of Zurich and Tumorzentrum Hirslanden Zurich, Zurich, Switzerland; 20 Mayo Clinic Florida, Jacksonville, FL USA; 21 Prostate Cancer Transatlantic Consortium, Jacksonville, FL USA; 22 Fox Chase Cancer Center – Temple Health, Philadelphia, PA USA; 23 Department of Epidemiology, Harvard T.H. Chan School of Public Health, Boston, MA USA

**Keywords:** Physical activity, Exercise, Resistance training, Advanced prostate cancer, Racial disparities, Regional disparities

## Abstract

**Purpose:**

To estimate guideline-based aerobic physical activity and resistance training participation in an international population of people with newly diagnosed metastatic hormone-sensitive or castration-resistant prostate cancer, and to assess racial and regional differences.

**Methods:**

Participants in the international IRONMAN registry (NCT 03151629) of advanced prostate cancer reported leisure-time activity at 6, 18, and 30 months post-enrolment. The proportion meeting guidelines of ≥ 150 min/week moderate to vigorous physical activity and ≥ 2 weekly resistance training sessions were quantified. Mixed-effects logistic regression estimated odds of meeting guidelines averaged across timepoints using all follow-up data by self-identified race (White, Black, Other) and geographic region (Europe, Australia, North America, Africa, Other).

**Results:**

Among 2485 participants, 58–63% met aerobic physical activity guidelines and 19–23% met resistance training guidelines across timepoints. Resistance training participation was especially low in African geographic regions (0–3%). Participants self-identifying as Black had lower odds of meeting aerobic physical activity guidelines than White participants (crude odds ratio [cOR] 0.23, 95% CI 0.15–0.36), and participants from Africa had lower odds than those from Europe (cOR 0.08, 95% CI 0.03–0.21). Crude patterns were similar for resistance training. Aerobic physical activity disparities persisted after multivariable adjustments, and in sensitivity analyses excluding Black participants from African geographic regions, while resistance training estimates were attenuated.

**Conclusions:**

Targeted strategies are warranted to increase participation rates in aerobic physical activity and resistance training among advanced prostate cancer patients self-identifying as Black, or those from African geographic regions.

**Supplementary Information:**

The online version contains supplementary material available at https://doi.org/10.1007/s00520-026-11251-3.

## Introduction

Prostate cancer is the second most common cancer among men worldwide, with more than 1.4 million new cases annually, and is the leading cause of cancer mortality in over 50 countries [1, 2]. Advanced prostate cancer includes disease that has metastasized to bone or visceral organs (metastatic hormone-sensitive prostate cancer, mHSPC) or cancer that progresses despite castrate testosterone levels (castration-resistant prostate cancer, CRPC), the latter accounting for most prostate cancer–related deaths. Advanced prostate cancer is currently incurable; treatment therefore focuses on slowing disease progression, alleviating symptoms, preserving quality of life, and reducing mortality risk [3].

Because most prostate tumors are driven by the androgen receptor pathway, the cornerstone of treatment is androgen suppression to castration levels, primarily through androgen deprivation therapy (ADT), often combined with androgen receptor pathway inhibitors (ARPI), chemotherapy, and other adjunct treatments [4]. While ADT is effective in reducing tumor burden, it is associated with significant adverse effects, including fatigue, psychological distress, increased fat mass, loss of lean body mass, and reduced physical function; factors that collectively impair quality of life and general health [5]. Critically, the incidence of advanced prostate cancer is expected to increase in the upcoming decades [1], and strategies to mitigate disease burden and treatment-related side effects are therefore warranted.

Lifestyle behaviors, particularly leisure-time physical activity for patients with prostate cancer, have gained considerable attention in recent years [6, 7]. Both aerobic physical activity and resistance training are safe and feasible for individuals with advanced prostate cancer, even among patients with bone metastases [8, 9]. Specifically, aerobic physical activity improves cardiometabolic health, while resistance training is particularly important to counter the ADT-related loss of lean body mass and muscle strength [9, 10], key factors linked to mortality and morbidity among those with advanced cancer types [11]. Furthermore, emerging evidence indicates a role of physical activity to improve treatment tolerance [7]. However, despite the growing evidence for the supportive role of physical activity, there is limited knowledge about the prevalence of meeting physical activity guidelines among individuals with advanced prostate cancer, whereas existing evidence is limited to small observational studies [12, 13].

A key issue in advanced prostate cancer research is inequity in outcomes among racially minoritized groups and individuals living in low- and middle-income countries. Patients who self-identify as Black experience poorer outcomes compared with White patients [14], and individuals in African geographic regions such as sub-Saharan Africa or the Caribbean face disproportionately higher mortality compared with those in high-income countries [14]. This diversity is poorly reflected in the physical activity and prostate cancer literature, and further research into physical activity among racial subgroups and low- to middle-income geographic regions has been warranted [15, 16]. This knowledge may contribute to identifying health and social inequities in supportive care and inform targeted interventions.

The present study aims to describe physical activity levels and the prevalence of meeting aerobic physical activity and resistance training guidelines in a large, international sample of patients with newly diagnosed advanced prostate cancer receiving routine care. Secondly, we aim to model average odds of meeting guidelines by race and geographic region subgroups.

## Methods

### Setting and study participants

**Fig. 1:**
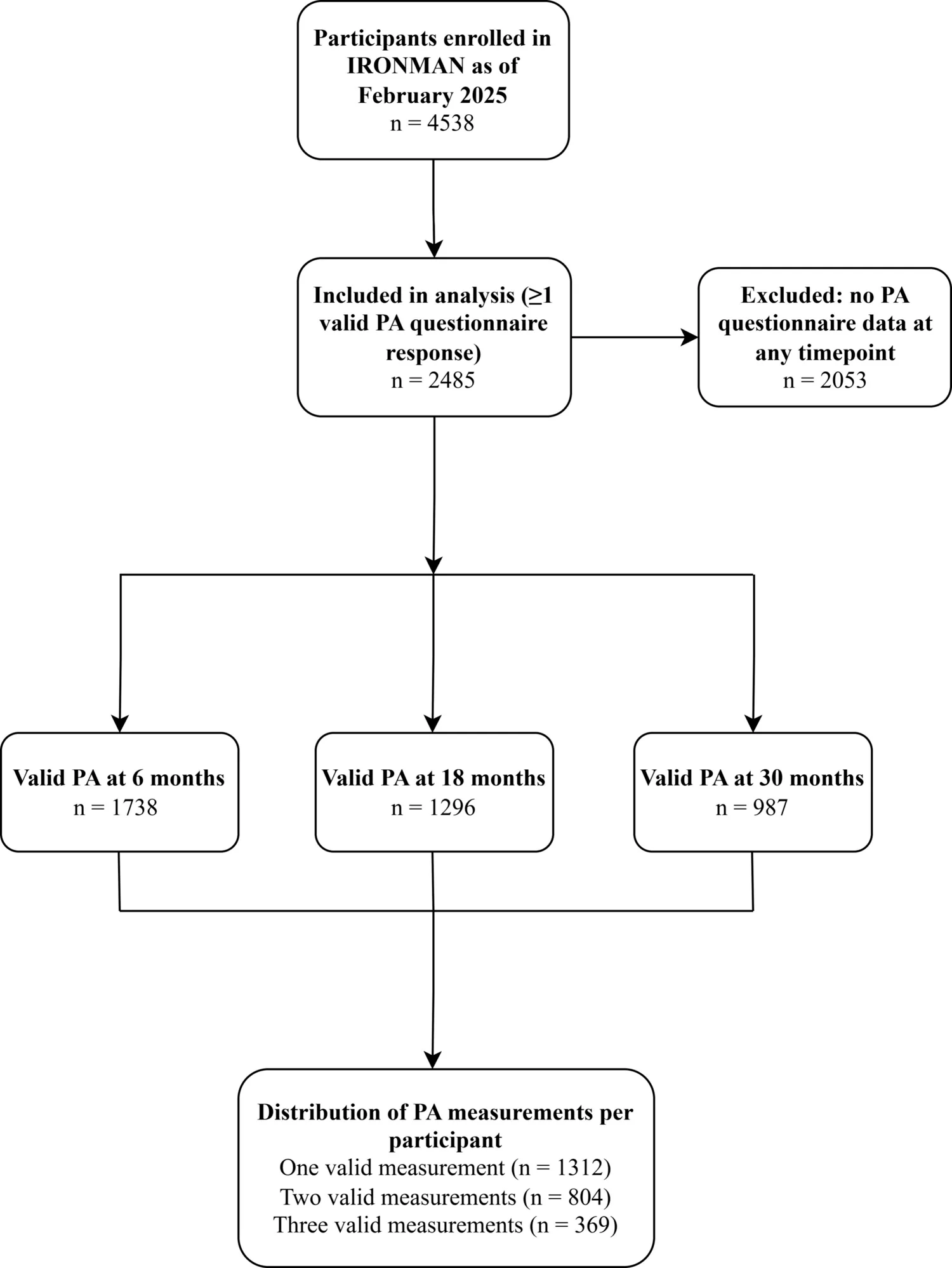
Flowchart showing inclusion of participants based on availability of physical activity (PA) questionnaire data in the IRONMAN registry. Participants were included in the analysis if they had at least one valid physical activity questionnaire response at 6, 18, or 30 months. Participants could contribute data at one or more timepoints. *Abbreviations*: n, number

This study used data from the International Registry for Men with Advanced Prostate Cancer (IRONMAN, ClinicalTrials.gov: NCT03151629, registered on May 15, 2017), a multinational registry initiated in 2017 across 15 countries representing high-, middle-, and low-income settings [17]. Eligible participants were aged ≥ 21 years, provided informed consent, and had a confirmed new diagnosis of mHSPC or CRPC, including de novo and recurrent metastatic prostate cancer. Individuals with recent non-prostate cancer requiring systemic therapy were excluded. Participants were followed for up to 5 years or until death, withdrawal, or loss to follow-up. The present analysis included participants with leisure-time physical activity from at least one follow-up assessment as of February 2025 (Fig. 1). The present study is reported in accordance with the STROBE guidelines for observational cohort studies [18] and follows Lesko et al.’s framework for descriptive epidemiology [19] (see Supplementary Materials).

### Measurements and procedures

#### Leisure-time physical activity levels

Physical activity was assessed at 6, 18, and 30 months after enrolment using the modified Paffenbarger physical activity questionnaire [20] (see Supplementary Materials for questionnaire details). The questionnaire collected data on time and intensity spent in various activities, including walking, jogging, running, cycling (indoor and outdoor), racquet sports, swimming, other aerobic exercises, and resistance exercise using arm and leg weights. Responses included predefined categories for duration (ranging from 0 min per week to ≥ 11 h per week), intensity (low, medium, high). Participants also self-reported walking speed using predefined categories (easy, normal, brisk, or very brisk), which was used to classify walking intensity.

All physical activity variables were derived independently for each timepoint. Total duration of time spent in aerobic physical activity and resistance training (derived from arm and leg resistance exercise items) was calculated using the middle value of the categorical response for each activity. Aerobic physical activities were classified as moderate or vigorous intensity based on established metabolic equivalent thresholds of ≥ 3.0 and ≥ 6.0, respectively, consistent with Pernar et al. [21]. Prevalence of meeting the 2019 American College of Sports Medicine exercise guidelines for cancer patients [22] was selected as the two primary outcomes, defined as

- Meeting aerobic physical activity guidelines of ≥ 150 min of moderate or ≥ 75 min of vigorous physical activity per week, or a combination.
- Meeting resistance training guidelines of at least two weekly sessions. Because the questionnaire did not capture resistance training frequency, we converted duration into sessions using a conservative cut-off of 15 min per session as a proxy for frequency, similar to other studies [23].

The questionnaire has shown acceptable validity for group-level moderate to vigorous physical activity estimates in epidemiological studies of middle-aged to older men (Spearman’s rho = 0.69) [21].

#### Demographic and clinical variables

Demographic and clinical variables were assessed at baseline (i.e., time of enrolment), including age (years), body mass index (BMI, kg/m^2^), marital status (married or living with partner/not married), education (higher [college or greater] vs. less than higher), and employment status (retired, full-time, not currently working). Health-related quality of life was assessed using the European Organisation for Research and Treatment of Cancer Quality of Life Questionnaire-Core 30 (EORTC QLQ-C30), summary score from 0 to 100 [24, 25]. Race is a social construct reflecting lived experiences and social conditions, which was self-identified using predefined categories. Categories were collapsed as Black, White, or Other (the latter reflecting small-number groups such as Asian or Middle Eastern). Geographic region was derived from the registered country of primary treatment site, grouped as Europe (England, Ireland, Norway, Spain, Sweden, Switzerland), North America (Canada, USA), Africa (Nigeria, Kenya, South Africa), Australia, and Other (Barbados, Jamaica, Brazil, due to small sample sizes of < 10 participants per country).

Clinical variables were extracted from medical records, physician questionnaires or blood specimens, including prostate-specific antigen (PSA, ng/mL), disease state (mHSPC/CRPC), and first on-study treatment (active treatments at days 0–90 post-enrolment: ADT + ARPI, ADT + ARPI + chemotherapy, ADT + chemo, or ADT monotherapy). Time on study was calculated from baseline to timepoint of last registered observation (months).

### Statistical analyses

Descriptive data are presented using median (interquartile ranges (IQR)) for continuous variables, and counts (percentages) for categorical variables. All physical activity durations were truncated at the 99th percentile to limit influence of extreme values, similar to other studies [26]. The crude prevalence of meeting the aerobic physical activity and resistance training guidelines at each timepoint was calculated overall and stratified by race, geographic region, disease state, and first on-study treatment regimen. Race and geographic region were included as stratifying variables due to evidence for disproportionate prostate cancer outcomes by these factors [14], and disease state and treatment regimen were included to provide clinical context.

We used mixed-effects logistic regression models using all available repeated measures data, to maximize statistical power to estimate the odds of meeting each guideline by race and geographic region subgroups across follow-up. Within-person change over time was not assessed due to uncertain responsiveness of the questionnaire. Meeting the guidelines was modelled as the dependent variable and race or geographic region as the primary independent variable. Models also included timepoint as a fixed effect and random intercepts for participants and treatment site to account for repeated measures and clustering. Analyses used maximum likelihood estimation under the assumption that outcome data were missing at random, which we assessed descriptively by comparing baseline characteristics across levels of data completeness.

We first fitted crude models for race and geographic region to reflect the unadjusted association of meeting guidelines [27]. Next, we created multivariable models adjusting for age, education, baseline ECOG status, and disease state and first on-study treatment regimen, variables known to be associated with physical activity levels [28, 29]. We did not perform mutual adjustments for race and geographic region due to conceptual overlap (most participants self-identifying as Black were from African sites), nor adjusted for other baseline variables that may be mediators rather than confounders between race/geographic region and physical activity. Missing data for categorical covariates were coded as “missing.” Subgroups with fewer than 10 participants were excluded due to limited sample size.

Sensitivity and exploratory analyses were performed to assess the robustness of the findings. These included (i) repeated analyses excluding month 30 and by restricting to participants with at least two valid timepoints (to evaluate influence of having missing data); (ii) excluding participant self-identifying as Black from the Africa geographic region in race analyses (to evaluate influence of region for racial analyses); (iii) stratified analyses by disease state (to detect potential differences between mHSPC and CRPC).

Results are reported as odds ratios (OR) with 95% confidence intervals (CI), relative to the reference level of each variable (herein defined as the subgroup with the greatest proportion meeting guidelines). An OR > 1 indicates higher odds of meeting guidelines in the comparison group than the reference group across timepoints. Statistical significance was evaluated at the 0.05 level. All analyses were conducted in R (version 4.4.1) using RStudio and the following packages: *tidyverse*, *lme4,* and *broom.mixed.*

## Results

Of the 4538 participants in the IRONMAN registry, 2485 responded to the leisure-time physical activity questionnaire at least once (Fig. 1). Compared to participants with no physical activity data, those with physical activity data were slightly younger, a greater proportion self-identified as White, had lower PSA levels, better EORTC QLQ-C30 summary scores, and a higher proportion had mHSPC at baseline (Supplementary Table S1). Participants with complete datasets had lower PSA levels, better ECOG performance status, and slightly higher EORTC QLQ-C30 summary scores than those with only one available response (Supplementary Table S2). Among participants, 75% had mHSPC and 25% had CRPC. The median age was approximately 70 years, and most participants self-identified as White (70%), and were primarily from North America (~40%) or Europe (~38%). EORTC QLQ-C30 summary scores were ~87/100 suggesting good quality of life. The most common first on-study treatment regimen was ADT + ARPI (Table 1). Descriptives by race and geographic region are provided in Supplementary Tables S3 and S4.

**Table 1:** Baseline characteristics of patients with advanced prostate cancer in IRONMAN Registry, stratified by disease state

Characteristic	mHSPC *N* = 1857	CRPC *N* = 624
Age (years)	69.0 (11.0)	72.0 (11.0)
BMI	26.7 (5.3)	28.0 (5.5)
Missing (*n*)	655	204
Geographic region		
Europe	696 (37%)	236 (38%)
Africa	231 (12%)	76 (12%)
Australia	121 (6.5%)	63 (10%)
North America	791 (43%)	232 (37%)
Other	18 (1.0%)	17 (2.7%)
Race		
White	1,291 (70%)	436 (70%)
Black	356 (19%)	117 (19%)
Other	210 (11%)	71 (11%)
Marital status		
Married or living with partner	1,371 (85%)	460 (87%)
Not married	246 (15%)	71 (13%)
Missing (*n*)	240	93
Employment status		
Working	482 (30%)	113 (21%)
Unemployed or Other	134 (8.4%)	50 (9.4%)
Retired	984 (62%)	367 (69%)
Missing (*n*)	257	94
Education level		
Higher education	646 (43%)	206 (41%)
Less than higher education	869 (57%)	293 (59%)
Missing (*n*)	342	125
EORTC QLQ-C30 summary score	88.5 (16.2)	86.6 (16.8)
Missing (*n*)	69	20
PSA (ng/mL)	5.10 (28.3)	5.89 (18.6)
Missing (*n*)	416	108
ECOG performance status		
0	832 (57%)	272 (59%)
1	462 (32%)	154 (33%)
2	105 (7.2%)	26 (5.7%)
3	50 (3.4%)	6 (1.3%)
4	11 (0.8%)	2 (0.4%)
Missing (*n*)	397	164
First on-study treatment regimen		
ADT + ARPI	906 (54%)	412 (75%)
ADT + ARPI + chemo	133 (8.0%)	31 (5.7%)
ADT + chemo	222 (13%)	38 (6.9%)
ADT monotherapy	410 (25%)	66 (12%)
Missing (*n*)	186	77
Time on study (months)	27 (27)	27 (30)

Continuous variables are presented as median (interquartile range), and categorical variables as counts (percentages). Four participants had missing disease state info at baseline and were not included in table

*BMI* body mass index, *EORTC QLQ-C30* European organisation for research and treatment of cancer quality of life questionnaire-core 30, *PSA* prostate-specific antigen, *ECOG* Eastern cooperative oncology group, *ADT* androgen deprivation therapy, *ARPI* androgen receptor pathway inhibitor, *Chemo* chemotherapy, *mHSPC* metastatic hormone-sensitive prostate cancer, *CRPC* castration-resistant prostate cancer

### Physical activity levels

Overall, median weekly duration of moderate to vigorous physical activity ranged from 190 to 210 min/week across timepoints, which was primarily composed of moderate-intensity activity (~ 150 min/week), with smaller contributions from vigorous activity (median 12–40 min/week). Median weekly resistance training was 0 min/week across all time points, whereas one-third of participants reported any resistance training at all (> 0 min/week). Prevalence of meeting aerobic physical activity guidelines ranged from ~58 to 63%, whereas 19–23% met resistance training guidelines in the overall sample (Table 2).

**Table 2:** Crude prevalence of meeting aerobic physical activity and resistance training guidelines at months 6, 18, and 30 overall and by race, geographic region, disease state, and treatment regimen

Guideline	Subgroup type	Subgroup	Month 6	Month 18	Month 30
Aerobic physical activity guidelines	Overall		1012/1738 (58%)	796/1296 (61%)	623/987 (63%)
	Race	White	774/1114 (69%)	634/926 (68%)	533/803 (66%)
		Black	104/417 (25%)	62/207 (30%)	31/77 (40%)
		Other	134/207 (65%)	100/163 (61%)	59/107 (55%)
	Geographic Region	Other	7/21 (33%)	6/13 (46%)	6/11 (55%)
		Europe	453/647 (70%)	343/514 (67%)	257/387 (66%)
		Africa	59/302 (20%)	27/122 (22%)	4/19 (21%)
		Australia	89/128 (70%)	64/103 (62%)	52/84 (62%)
		North America	404/640 (63%)	356/544 (65%)	304/486 (63%)
	Disease state	mHSPC	784/1325 (59%)	621/984 (63%)	474/727 (65%)
		CRPC	225/409 (55%)	174/311 (56%)	149/260 (57%)
	First on-study treatment regimen	ADT + ARPI	539/805 (67%)	419/644 (65%)	317/492 (64%)
		ADT + ARPI + chemo	99/137 (72%)	56/74 (76%)	16/23 (70%)
		ADT + chemo	80/116 (69%)	84/118 (71%)	80/125 (64%)
		ADT monotherapy	107/202 (53%)	85/151 (56%)	82/126 (65%)
Resistance training	Overall		325/1694 (19%)	279/1279 (22%)	220/969 (23%)
	Race	White	259/1088 (24%)	233/912 (26%)	193/788 (24%)
		Black	29/412 (7%)	19/207 (9%)	14/76 (18%)
		Other	37/194 (19%)	27/160 (17%)	13/105 (12%)
	Geographic region	Other	0/21 (0%)	0/13 (0%)	1/11 (9%)
		Europe	125/616 (20%)	108/500 (22%)	72/375 (19%)
		Africa	9/300 (3%)	1/122 (1%)	0/18 (0%)
		Australia	37/127 (29%)	29/103 (28%)	27/84 (32%)
		North America	154/630 (24%)	141/541 (26%)	120/481 (25%)
	Disease state	mHSPC	262/1296 (20%)	232/972 (24%)	170/712 (24%)
		CRPC	61/394 (15%)	46/306 (15%)	50/257 (19%)
	First on-study treatment regimen	ADT + ARPI	173/776 (22%)	161/634 (25%)	118/482 (24%)
		ADT + ARPI + chemo	38/136 (28%)	24/74 (32%)	5/23 (22%)
		ADT + chemo	20/109 (18%)	23/115 (20%)	29/123 (24%)
		ADT monotherapy	30/201 (15%)	30/150 (20%)	28/125 (22%)

Numbers indicate crude prevalence rates per timepoint derived from the number of available participants at each timepoint

*PA* physical activity, *mHSPC* metastatic hormone-sensitive prostate cancer, *CRPC* castration-resistant prostate cancer, *ADT* androgen deprivation therapy, *ARPI* androgen receptor pathway inhibitor, *chemo* chemotherapy

Crude prevalence of meeting aerobic physical activity guidelines was consistently higher among participants who self-identified as White compared with Black, and in participants from Europe, Australia, and North America than Africa and Other regions (for example, month 6: White 69% vs Black 25%; Europe 70%, Australia 70%, North America 63% vs Africa 20%) (Table 2). Prevalence of meeting resistance training guidelines was markedly lower overall, and especially scarce in participants from Africa (0–3%) and Other geographic regions (0–9%) compared to Europe, Australia, and North America (19–32%) (Table 2). Participants with mHSPC had higher prevalence of meeting both guidelines than CRPC, and those receiving ADT monotherapy had lower prevalence than those treated using other regimens (Table 2).

### Odds of meeting guidelines in racial and regional subgroups

Odds of meeting aerobic physical activity guidelines were lower among participants self-identifying as Black compared with participants self-identifying as White (crude OR [cOR] 0.23, 95% CI 0.15–0.36). This disparity persisted after adjustment for demographic and clinical factors (Table 3), as well as in sensitivity analyses excluding participants self-identifying as Black from African (leaving 181 participants in the Black subgroup) (Supplementary Table S5). Participants categorized as Other race also had lower odds of meeting aerobic physical activity guidelines than participants self-identifying as White (Table 3). For meeting resistance training guidelines, participants self-identifying as Black had lower crude odds (cOR 0.30, 95% CI 0.10–0.89), but this association was not statistically significant after adjustment (Table 3) or when excluding participants self-identifying as Black from African geographic regions (Supplementary Table S5). Finally, participants from African geographic regions had lower crude odds of meeting both aerobic physical activity and resistance training guidelines compared with those from Europe (cOR aerobic physical activity 0.08, 95% CI 0.03–0.21; cOR resistance training 0.12, 95% CI 0.02–0.97 (Table 3)).

**Table 3:** Findings from mixed-effects logistic regression for odds of meeting aerobic physical activity and resistance training guidelines over time

		Aerobic physical activity guidelines		Resistance training guidelines	
Subgroup type	Subgroup	cOR (95% CI)	aOR (95% CI)	cOR (95% CI)	aOR (95% CI)
Race (ref = White)	Black	**0.23 (0.15–0.36)**	**0.25 (0.17–0.39)**	**0.30 (0.1–0.89)**	0.45 (0.14–1.48)
	Other	**0.67 (0.49–0.93)**	**0.68 (0.49–0.94)**	0.62 (0.22–1.76)	0.60 (0.2–1.79)
Geographic region (ref = Europe)	Australia	1.18 (0.56–2.51)	1.29 (0.62–2.71)	1.58 (0.51–4.87)	1.86 (0.56–6.2)
	North America	0.82 (0.51–1.32)	0.82 (0.51–1.31)	1.29 (0.65–2.53)	1.25 (0.62–2.51)
	Africa	**0.08 (0.03–0.21)**	**0.16 (0.06–0.4)**	**0.12 (0.02–0.97)**	0.18 (0.02–1.72)

Odds ratios (OR) and 95% confidence intervals (CI) are shown for subgroup comparisons. An OR > 1 indicates higher odds of meeting guidelines compared to the reference subgroup across timepoints. Crude odds ratios (cOR) were adjusted for timepoint (fixed effects) and include a random intercept for the participant to account for within-subject variation and study site for clustering. Adjusted odds ratios (aOR) were further adjusted for age, education, ECOG status, disease state and first on-study treatment. Statistically significant (*p* < 0.05) odds ratios are highlighted with **bold** font

*ref* reference

Sensitivity analyses excluding month 30 and excluding participants with less than two valid timepoints produced estimates similar to the main analysis (Supplementary Tables S6 and S7). Stratification by disease state showed that racial and regional disparities persisted among participants with mHSPC for aerobic physical activity, while associations were attenuated among participants with CRPC (Supplementary Table S8).

## Discussion

To the authors’ best knowledge, the present study is the largest study to describe prevalence of meeting aerobic physical activity and resistance training guidelines in an internationally diverse cohort of men with advanced prostate cancer. We found that despite the advanced state of their cancers, there was good prevalence of meeting aerobic physical activity guidelines (58–63%), although lower prevalence was seen for meeting resistance training guidelines (19–23%). Odds of meeting aerobic physical activity guidelines were lower for participants self-identifying as Black compared to White and for participants from Africa compared to Europe, while the crude prevalence of meeting resistance training guidelines was especially low in participants from Africa and the Other geographic region.

Our findings on aerobic physical activity guidelines are partly consistent with other studies focusing on various stages of prostate cancer, which report prevalence rates ranging from 21 to 73%. Two previous studies in 140 and 55 patients with advanced prostate cancer from high-income countries, respectively, reported that only 29% met aerobic physical activity guidelines, which is lower than the 58–63% observed in our study [12, 13]. These studies did, however, exclude participants meeting physical activity guidelines and used a different questionnaire than the present study. Nevertheless, our inclusion of a broad and diverse sample without exclusion based on physical activity levels suggests that the prevalence of meeting aerobic physical activity guidelines might be higher than 29% among men with advanced prostate cancer.

In studies focusing on localized prostate cancer, 21–73% and 13–30% meet aerobic physical activity and resistance training guidelines, respectively [12, 30–36]. Our estimates for advanced prostate cancer fall within these ranges, although differing participants characteristics and use of different physical activity questionnaires make it challenging to compare estimates directly. However, in a study of mixed cancer survivors assessed with a questionnaire comparable to ours, 66% met aerobic physical activity guidelines [37], which is higher than the participants in our study.

A notable finding was that the prevalence of meeting resistance training guidelines was much lower than for aerobic physical activity. Resistance training, as operationalized in the present study using free weights or machines, typically requires greater access to facilities, equipment, or supervision than aerobic physical activities such as walking. Ideally, however, the proportion meeting resistance training in this population should be higher, given its important role in limiting ADT-related loss of muscle mass and reduced physical function [38, 39], and studies linking resistance training participation to reduced cancer mortality [23, 40].

We observed lower crude odds of meeting aerobic physical activity guidelines among participants self-identifying as Black versus White participants, with 70–77% lower crude odds for both aerobic physical activity and resistance training. This disparity may reflect social and health-system factors including socioeconomic status, health literacy, or access to supportive care, rather than biological differences or individual motivation [41]. Findings were similar in sensitivity analyses excluding Black participants from African geographic regions, suggesting that geographic region alone does not explain lower activity levels. Our findings align with Asare et al., who reported 23% lower odds of meeting aerobic physical activity guidelines among cancer survivors identifying as Black versus White, although the magnitude was smaller than observed here [42]. Given the persistent racial disparities in prostate cancer outcomes, inequities in access to and support for lifestyle behaviors such as leisure-time physical activity may contribute to these differences [14]. Differences by geographic region were also observed; participants from Africa and Other geographic regions had lower prevalence of meeting both physical activity and resistance training guidelines, with resistance training being nearly absent in Africa (0–3%). This contrasts with global data showing higher overall physical activity levels in some African countries [43], likely because our questionnaire captures leisure-time activity and excludes occupational, transport, and household domains that contribute substantially to total activity in many low- and middle-income countries [43]. Limited access to safe environments, facilities, equipment, and support may further constrain participation [16]. In addition, the resistance training questionnaire items may not reflect locally available or culturally typical strength activities, potentially leading to under-reporting. Limited implementation and reimbursement opportunities for exercise oncology in Africa have also been reported previously, consistent with our findings [16].

Participants with mHSPC had slightly higher prevalence of meeting both guidelines than those with CRPC. One study showed that exercise levels among participants with mHSPC and CRPC are comparable when receiving supervised exercise [44]. However, patients with CRPC may have higher disease and treatment burden, which may make it more challenging to be physically active. Participants primarily treated with ADT monotherapy reported lower prevalence of meeting both guidelines. These patients may have poorer physical performance status and are less capable of more intensive treatment combinations, or cross-country differences in treatment availability and protocols.

### Strengths and limitations

A key strength of this study is the inclusion of a large, international, and racially diverse sample, including participants from low- and middle-income countries, which is rare in oncology research [16]. Notably, the IRONMAN registry applies broad inclusion criteria and has a low non-acceptance rate, which facilitates strong representativeness of the population with advanced prostate cancer. The availability of longitudinally collected data on physical activity is also a strength as it enabled greater statistical power and reduced bias from single timepoint measurements [45]. Next, by using mixed-effects models to utilize all available data from multiple follow-ups, we were able to increase statistical power for subgroup analyses. Finally, we considered a range of potential stratifying variables to show existing disparities in the uptake of physical activity as supportive care opportunities using international physical activity recommendations that enable comparison to other studies.

The present study also has important limitations. Although IRONMAN has a high acceptance rate, the participants providing answers to the physical activity questionnaire were slightly younger, had lower PSA and ECOG status, and higher proportion of mHSPC compared to CRPC than those not answering the physical activity questionnaire. Hence, prevalence rates may be overestimated relative to the full IRONMAN population due to more healthy respondents than non-respondents. Next, we lacked sufficiently complete comorbidity data, which is relevant for the participants’ ability to be active. However, analyses were adjusted for ECOG performance status, which should capture functional limitations due to comorbidities.

The long recall period and reliance on self-reporting for the physical activity questionnaire may have led to recall and social desirability bias, which may have overestimated the prevalence of meeting guidelines [46]. While we used a validated instrument [21], misclassification of intensity and duration cannot be ruled out, and interpretation may vary across cultural and regional contexts [47]. Finally, resistance training estimates were based on duration as a proxy for frequency, where the selected cut-off (15 min = 1 session) may bias the observed absolute prevalence rate.

Real-world clinical epidemiological data is often subject to missingness, and this study was no exception, with month 30 being especially scarce in some subgroups such as the African geographic region. Hence, even though the mixed-effects model accounted for missing outcome data, missingness may still influence results. However, sensitivity analyses suggest that our findings were robust to missing data.

## Conclusion

In the present study, the prevalence of meeting aerobic physical activity guidelines was approximately three times as higher than that of meeting the resistance training guidelines among men with advanced prostate cancer. Participants self-identifying as Black and participants from African geographic regions had significantly lower odds of meeting aerobic physical activity guidelines compared to those self-identifying as White or from Europe. Notably, we also observed markedly lower prevalence of meeting resistance training guidelines in these subgroups, with prevalence in Africa being especially low. These findings indicate a potential for developing strategies that address structural and cultural barriers and integrate physical activity support into routine care. Moreover, interventions should focus on being accessible and culturally appropriate for high-risk subgroups such as those identified in the present study.

## Supplementary Information

Below is the link to the electronic supplementary material.

ESM 1 (DOCX 849 KB)

## Data Availability

The data generated in this study are not publicly available due to privacy of research participants but are available upon reasonable request and approval of the IRONMAN Executive Committee.
