# Correction to: Prevalence of meeting aerobic physical activity and resistance training guidelines in advanced prostate cancer: a descriptive study of racial and regional disparities in the IRONMAN registry

**DOI:** 10.1007/s00520-026-11294-6

**Published:** 2026-10-02

**Authors:** Aleksander Solberg, May Grydeland, Tormod S. Nilsen, Anne H. Storås, Ian D. Davis, Natalie Greaves, Simon Anderson, Andre P. Fay, Kim N. Chi, Deborah Enting, Raymond McDermott, Simone Badal, Charles G. Waihenya, Ademola Popoola, John M. Lazarus, Joaquin Mateo, Anders Bjartell, Aurelius Omlin, Folakemi Odedina, Camille Ragin, Lorelei A. Mucci, Kjell M. Russnes

**Affiliations:** 1 Department of Physical Performance, The Norwegian School of Sport Sciences, Oslo, Norway; 2 Department of Sport Science and Physical Education, University of Agder, Kristiansand, Norway; 3 Department of Oncology, Oslo University Hospital, Oslo, Norway; 4 Eastern Health Clinical School, Monash University, Melbourne, Australia; 5 Cancer Services, Eastern Health, Melbourne, Australia; 6 George Alleyne Chronic Disease Research Centre, The University of the West Indies, Cave Hill Campus, Bridgetown, Barbados; 7 African Caribbean Cancer Consortium, Philadelphia, USA; 8 PUCRS School of Medicine, Hospital Nora Teixeira, Santa Casa de Porto Alegre, Porto Alegre, Brazil; 9 BC Cancer, Vancouver Prostate Centre, Department of Medicine, University of British Columbia, Vancouver, Canada; 10 Department of Oncology, Guy’s and St Thomas’ NHS Foundation Trust, London, UK; 11 St. Vincent’s University Hospital & Cancer Trials, Dublin, Ireland; 12 Department of Basic Medical Sciences, The University of the West Indies, Kingston, Jamaica; 13 Department of Surgery, University of Nairobi, Nairobi, Kenya; 14 Department of Urology, University of Ilorin Teaching Hospital, Ilorin, Nigeria; 15 Prostate Cancer Transatlantic Consortium, Ilorin, Nigeria; 16 Department of Urology, Groote Schuur Hospital, University of Cape Town, Cape Town, South Africa; 17 Vall d’Hebron Institute of Oncology, Barcelona, Spain; 18 Department of Translational Medicine (Urology), Skåne University Hospital, Lund University, Lund, Sweden; 19 Onkozentrum Zurich, University of Zurich and Tumorzentrum Hirslanden Zurich, Zurich, Switzerland; 20 Mayo Clinic Florida, Jacksonville, Florida USA; 21 Prostate Cancer Transatlantic Consortium, Jacksonville, Florida USA; 22 Fox Chase Cancer Center – Temple Health, Philadelphia, Pennsylvania USA; 23 Department of Epidemiology, Harvard T.H. Chan School of Public Health, Boston, MA USA

**Correction to: Supportive Care in Cancer (2026)**

**https://doi.org/10.1007/s00520-026-11251-3**

In the published article, the descriptive reported median duration of moderate-intensity activity performed per week was incorrect. The reported amount was 50 minutes per week, but the correct amount is 150 minutes per week. The following sentence in the Results section was incorrectly reported:

- “Overall, median weekly duration of moderate to vigorous physical activity ranged from 190 to 210 min/week across timepoints, which was primarily composed of moderate-intensity activity (~ 50 min/week), with smaller contributions from vigorous activity (median 12–40 min/week).”

The sentence should read:

- “Overall, median weekly duration of moderate to vigorous physical activity ranged from 190 to 210 min/week across timepoints, which was primarily composed of moderate-intensity activity (~ 150 min/week), with smaller contributions from vigorous activity (median 12–40 min/week).”

This correction does not affect the analyses, interpretation, or conclusions of the article.

The original article has been corrected.

