# Supplementary material for: Prevalence of meeting aerobic physical activity and resistance training guidelines in advanced prostate cancer: a descriptive study of racial and regional disparities in the IRONMAN registry
Source: Support Care Cancer. 2026 Sep 22;34(10):998. doi: 10.1007/s00520-026-11251-3 (PMC13597563; doi:10.1007/s00520-026-11251-3)
Supplement: Supplementary file 1 [file 520_2026_11251_MOESM1_ESM.docx]

# Supplemental Materials

**Journal:** Supportive care in cancer

## Title: Prevalence of Meeting Physical Activity and Resistance Training Guidelines in Advanced Prostate Cancer: A Descriptive Study of Racial and Regional Inequities in the IRONMAN Registry

## Author list: Aleksander Solberg¹, May Grydeland¹, Tormod S. Nilsen¹^,^², Anne H. Storås^3^, Ian D. Davis^4,5^, Natalie Greaves^6^, Simon Anderson^6^, Andre P. Fay^7^, Kim N. Chi^8^, Deborah Enting^9^, Raymond McDermott^10^, Simone Badal^11^, Charles G. Waihenya^12^, Ademola Popoola^13,14^, John M. Lazarus^15^, Joaquin Mateo^16^, Anders Bjartell^17^, Aurelius Omlin^18^, Folakemi Odedina^19,20^, Camille Ragin^21^, Lorelei A. Mucci^22^ on behalf of the IRONMAN investigators, Kjell M. Russnes^3^

## Affiliations

1. Department of Physical Performance, The Norwegian School of Sport Sciences, Oslo, Norway.
2. Department of Sport Science and Physical Education, University of Agder, Kristiansand, Norway
3. Department of Oncology, Oslo University Hospital, Oslo, Norway
4. Eastern Health Clinical School, Monash University, Melbourne, Australia
5. Cancer Services, Eastern Health, Melbourne, Australia
6. George Alleyne Chronic Disease Research Centre, The University of the West Indies, Cave Hill Campus, Bridgetown, Barbados
7. PUCRS School of Medicine, Hospital Nora Teixeira, Santa Casa de Porto Alegre, Porto Algere, Brazil
8. BC Cancer, Vancouver Prostate Centre, Department of Medicine, University of British Columbia, Vancouver, Canada
9. Department of Oncology, Guy’s and St Thomas’ NHS Foundation Trust, London, UK
10. St. Vincents University Hospital & Cancer Trials, Dublin, Ireland
11. Department of Basic Medical Sciences, The University of the West Indies, Kingston, Jamaica
12. Department of Surgery, University of Nairobi; Nairobi, Kenya
13. Department of Urology, University of Ilorin Teaching Hospital, Ilorin, Nigeria
14. Prostate Cancer Transatlantic Consortium, Ilorin, Nigeria
15. Department of Urology, Groote Schuur Hospital, University of Cape Town, Cape Town, South Africa
16. Vall d’Hebron Institute of Oncology, Barcelona, Spain
17. Department of Translational Medicine (Urology), Skåne University Hospital, Lund University, Lund, Sweden
18. Onkozentrum Zurich, University of Zurich and Tumorzentrum Hirslanden Zurich, Zurich, Switzerland
19. Mayo Clinic Florida, Jacksonville, Florida, USA
20. Prostate Cancer Transatlantic Consortium, Jacksonville, Florida, USA
21. Fox Chase Cancer Center – Temple Health, Philadelphia, Pennsylvania, USA
22. Department of Epidemiology, Harvard T.H. Chan School of Public Health, Boston, MA, USA

**Corresponding author:** Aleksander Solberg (aleksandso@nih.no)

## STROBE checklist

STROBE Statement—Checklist of items that should be included in reports of ***cohort studies***

|  | Item No | Recommendation |  |
| --- | --- | --- | --- |
| **Title and abstract** | 1 | (*a*) Indicate the study’s design with a commonly used term in the title or the abstract | X |
|  |  | (*b*) Provide in the abstract an informative and balanced summary of what was done and what was found | X |
| Introduction | | |  |
| Background/rationale | 2 | Explain the scientific background and rationale for the investigation being reported | X |
| Objectives | 3 | State specific objectives, including any prespecified hypotheses | X |
| Methods | | |  |
| Study design | 4 | Present key elements of study design early in the paper | X |
| Setting | 5 | Describe the setting, locations, and relevant dates, including periods of recruitment, exposure, follow-up, and data collection | X |
| Participants | 6 | (*a*) Give the eligibility criteria, and the sources and methods of selection of participants. Describe methods of follow-up | X |
|  |  | (*b*) For matched studies, give matching criteria and number of exposed and unexposed | Not relevant |
| Variables | 7 | Clearly define all outcomes, exposures, predictors, potential confounders, and effect modifiers. Give diagnostic criteria, if applicable |  |
| Data sources/ measurement | 8* | For each variable of interest, give sources of data and details of methods of assessment (measurement). Describe comparability of assessment methods if there is more than one group | *X* |
| Bias | 9 | Describe any efforts to address potential sources of bias | X |
| Study size | 10 | Explain how the study size was arrived at | X |
| Quantitative variables | 11 | Explain how quantitative variables were handled in the analyses. If applicable, describe which groupings were chosen and why | X |
| Statistical methods | 12 | (*a*) Describe all statistical methods, including those used to control for confounding | X |
|  |  | (*b*) Describe any methods used to examine subgroups and interactions | X |
|  |  | (*c*) Explain how missing data were addressed | X |
|  |  | (*d*) If applicable, explain how loss to follow-up was addressed | Not applicable |
|  |  | (*e*) Describe any sensitivity analyses | X |
| Results | | |  |
| Participants | 13* | (a) Report numbers of individuals at each stage of study—eg numbers potentially eligible, examined for eligibility, confirmed eligible, included in the study, completing follow-up, and analysed | X |
|  |  | (b) Give reasons for non-participation at each stage |  |
|  |  | (c) Consider use of a flow diagram | X |
| Descriptive data | 14* | (a) Give characteristics of study participants (eg demographic, clinical, social) and information on exposures and potential confounders | X |
|  |  | (b) Indicate number of participants with missing data for each variable of interest | X (Table 2) |
|  |  | (c) Summarise follow-up time (eg, average and total amount) | X |
| Outcome data | 15* | Report numbers of outcome events or summary measures over time | X |
| Main results | 16 | (*a*) Give unadjusted estimates and, if applicable, confounder-adjusted estimates and their precision (eg, 95% confidence interval). Make clear which confounders were adjusted for and why they were included | X |
|  |  | (*b*) Report category boundaries when continuous variables were categorized | X |
|  |  | (*c*) If relevant, consider translating estimates of relative risk into absolute risk for a meaningful time period | X |
| Other analyses | 17 | Report other analyses done—eg analyses of subgroups and interactions, and sensitivity analyses | X |
| Discussion | | |  |
| Key results | 18 | Summarise key results with reference to study objectives | X |
| Limitations | 19 | Discuss limitations of the study, taking into account sources of potential bias or imprecision. Discuss both direction and magnitude of any potential bias | X |
| Interpretation | 20 | Give a cautious overall interpretation of results considering objectives, limitations, multiplicity of analyses, results from similar studies, and other relevant evidence | X |
| Generalisability | 21 | Discuss the generalisability (external validity) of the study results | X |
| Other information | | |  |
| Funding | 22 | Give the source of funding and the role of the funders for the present study and, if applicable, for the original study on which the present article is based | X |

## Framework for descriptive studies (Framework by Lesko et al. 2022 Am J Epidemiol.)

| **Article Section and Item** | **Item No.** | **Recommendation(s)** | **Page no.** |
| --- | --- | --- | --- |
| Title and abstract | 1 | Explicitly state that this is a “descriptive study” in the title or the abstract. | Title |
|  | 2 | Summarize the target population and provide an informative and balanced summary of estimated disease occurrence in the abstract. | Abstract |
| Introduction |  |  |  |
| Background/rationale | 3 | State the motivation for the study, including, where relevant, the action that might be informed by the results. | 2 |
| Objectives | 4 | State the descriptive estimand, explicitly including: (a) the target population (who would be affected by any decisions made as a result of the study?); (b) the health state to be summarized; (c) the measure of occurrence; and (d) any stratification variables, if applicable. | 2-3 |
| Methods |  |  |  |
| Study design | 5 | (a) State whether the study is cross-sectional or longitudinal. (b) Restate the measure of occurrence being targeted. (c) If the study is longitudinal, specify the time origin and follow-up period for the measure of occurrence; if the study is cross-sectional, specify the time anchor at which the health state is summarized for individuals. | 3/4 |
| Setting | 6 | Describe any relevant features of the place and time in which the target population resides and across which data were collected. | 3 |
| Participants | 7 | (a) Describe the target population thoroughly in terms of person, place, and time. (b) Describe sampling into the study population (whether sampling was explicit or implicit, e.g., by inclusion in an administrative database); this includes eligibility criteria (see recommendations on data sources in item 10 below). (c) Describe any restrictions on the analytical sample. | 3 |
| Outcome(s) | 8 | (a) State when and how the outcome is measured. (b) Include estimates or discussion of the sensitivity and specificity of the study outcome definition relative to the gold standard. (c) List secondary outcomes or competing events of interest. | 3/4 |
| Covariates | 9 | Specify any stratification or adjustment variables—clearly define how variables were collected or constructed. | 4/5 |
| Data sources/measurement | 10 | Clearly delineate any inclusion/exclusion criteria for membership in the data source, including the original purpose for which the data were collected, if not for the study at hand. | 3 |
| Bias | 11 | Describe any assumptions or methods used to extrapolate data from the analytical sample to the study population and from the study population to the target population. | 4 |
| Statistical methods | 12 | (a) Describe the primary statistical methods used to estimate the measure of disease occurrence being targeted; discuss assumptions of that method in light of data limitations (e.g., assumption of independent censoring for people lost to follow-up). (b) If any adjustment/standardization will be done, state the goal of such adjustment. | 5 |
| Results |  |  |  |
| Participants | 13 | Report numbers of individuals at each study stage (this is likely to be approximate for the target population); consider summarizing this information in a flow diagram. | Figure 1 |
| Descriptive data | 14 | (a) Report on the characteristics of the analytical sample in a “Table 1.” (b) Indicate the number of participants with missing data for each variable used in the analysis. (c) If any weighting or imputation is done to reconstruct the study sample or target populations, include columns for those populations. | Table 1, S1,2,3,4 |
| Outcome data | 15 | (a) Present an overall (unstratified) estimate of the measure of occurrence of interest. (b) Report “crude” (raw data in the analytical sample) and (if applicable) “corrected” (after any weighting or imputation) estimates. | Table 2 |
| Other analyses | 16 | Present prespecified stratum-specific or adjusted/standardized results. | Table 3 |
| Discussion |  |  |  |
| Key results | 17 | Summarize key results with reference to the study objectives. | 8 |
| Limitations | 18 | Summarize potential sources of selection bias and measurement error and any attempts to mitigate these biases. Discuss both the direction and magnitude of any potential bias. Integrating quantitative bias analysis into the study to guide these discussions is encouraged. | 11 |
| Interpretation | 19 | (a) Avoid causal interpretations of descriptive results; avoid overinterpreting stratum-specific differences in measures of occurrence. (b) Describe how results of this study might inform or improve public health or clinical practice. | 8-10 |

## Physical activity questionnaire details

### Classification of aerobic physical activities

| Moderate Activities (3.0–5.9 METs*) | Vigorous Activities (>6 METs*) |
| --- | --- |
| Walking (self-reported pace 2.0–3.9 mph / 3.2–6.2 km/h) | Walking (≥4 mph / ≥6.4 km/h), climbing stairs |
| Lower-intensity exercise | Jogging, running |
| Moderate/heavy outdoor work | High-intensity bicycling, lap swimming, tennis, squash or racquetball |
| Other aerobic exercise | Other vigorous activities |
|  |  |
| Low or moderate intensity bicycling, lap swimming, tennis, squash or racquetball |  |

*Assigned based on the 2011 compendium of physical activities. Abbreviations: METs: metabolic equivalent of task; mph = miles per hour; km/h = kilometres per hour

### Middle values used to estimate duration of each reported activity

| Category | Middle value (minutes) |
| --- | --- |
| Zero | 0.0 |
| 1–4 Minutes | 2.5 |
| 5–19 Minutes | 12.0 |
| 20–59 Minutes | 39.5 |
| One Hour | 60.0 |
| 1–1.5 Hours | 75.0 |
| 2–3 Hours | 150.0 |
| 4–6 Hours | 300.0 |
| 7–10 Hours | 510.0 |
| 11+ Hours | 660 |

Supplementary Table S1. Baseline characteristics of patients with advanced prostate cancer in IRONMAN Registry, stratified by availability of at least one physical activity questionnaire response

| **Characteristic** | **Invalid**  N = 2,053^1^ | **Valid**  N = 2,485^1^ |
| --- | --- | --- |
| **Age (years)** | 71.0 (12.0) | 70.0 (11.0) |
| Missing (n) | 552 | 0 |
| **BMI** | 27.1 (6.2) | 27.0 (5.5) |
| Missing (n) | 1,143 | 860 |
| **Geographic Region** |  |  |
| Europe | 538 (36%) | 933 (38%) |
| Africa | 202 (13%) | 307 (12%) |
| Australia | 70 (4.6%) | 187 (7.5%) |
| North America | 645 (43%) | 1,023 (41%) |
| Other | 55 (3.6%) | 35 (1.4%) |
| Missing (n) | 543 | 0 |
| **Race** |  |  |
| White | 952 (63%) | 1,729 (70%) |
| Black | 336 (22%) | 473 (19%) |
| Other | 222 (15%) | 283 (11%) |
| Missing (n) | 543 | 0 |
| **Marital Status** |  |  |
| Married or Living With Partner | 1,056 (83%) | 1,835 (85%) |
| Not Married | 218 (17%) | 317 (15%) |
| Missing (n) | 779 | 333 |
| **Employment Status** |  |  |
| Working | 328 (26%) | 596 (28%) |
| Unemployed or Other | 114 (9.0%) | 184 (8.6%) |
| Retired | 828 (65%) | 1,354 (63%) |
| Missing (n) | 783 | 351 |
| **Education Level** |  |  |
| Higher Education | 468 (40%) | 853 (42%) |
| Less than higher education | 690 (60%) | 1,165 (58%) |
| Missing (n) | 895 | 467 |
| **EORTC QLQ-C30 Summary Score** | 85.6 (18.5) | 87.9 (16.3) |
| Missing (n) | 654 | 90 |
| **PSA (ng/mL)** | 9.50 (45.00) | 5.50 (25.76) |
| Missing (n) | 518 | 527 |
| **ECOG Performance Status** |  |  |
| 0 | 838 (53%) | 1,105 (58%) |
| 1 | 582 (36%) | 616 (32%) |
| 2 | 119 (7.5%) | 131 (6.8%) |
| 3 | 45 (2.8%) | 56 (2.9%) |
| 4 | 12 (0.8%) | 13 (0.7%) |
| Missing (n) | 457 | 564 |
| **Disease State** |  |  |
| mHSPC | 1,334 (66%) | 1,857 (75%) |
| CRPC | 681 (34%) | 624 (25%) |
| Missing (n) | 38 | 4 |
| **First On-Study Treatment Regimen** |  |  |
| ADT + ARPI | 922 (55%) | 1,319 (59%) |
| ADT + ARPI + chemo | 94 (5.6%) | 164 (7.4%) |
| ADT + chemo | 222 (13%) | 260 (12%) |
| ADT monotherapy | 437 (26%) | 476 (21%) |
| Missing (n) | 378 | 266 |
| **Time on Study (Months)** | 12 (21) | 27 (27) |
| Missing (n) | 0 | 0 |

Continuous variables are presented as median (interquartile range), and categorical variables as counts (percentages). Abbreviations: BMI: Body Mass Index; EORTC QLQ-C30: European Organisation for Research and Treatment of Cancer Quality of Life Questionnaire-Core 30; PSA: Prostate-Specific Antigen; ECOG: Eastern Cooperative Oncology Group; ADT: Androgen Deprivation Therapy; ARPI: Androgen Receptor Pathway Inhibitor; Chemo: Chemotherapy; mHSPC: Metastatic Hormone-Sensitive Prostate Cancer; CRPC: Castration-Resistant Prostate Cancer.

Supplemental Table S2. Baseline Characteristics by Number of Available Physical Activity Questionnaire Data at Follow-Ups

| **Characteristic** | **One valid measurement**  N = 1,315 | **Two valid measurements**  N = 744 | **Three valid measurements**  N = 346 |
| --- | --- | --- | --- |
| **Age (years)** | 70.0 (12.0) | 70.0 (11.0) | 70.0 (11.0) |
| **BMI** | 27.0 (5.6) | 26.6 (5.2) | 27.6 (5.7) |
| Missing (n) | 472 | 229 | 90 |
| **Geographic Region** |  |  |  |
| Europe | 465 (35%) | 301 (41%) | 141 (41%) |
| Africa | 190 (14%) | 95 (13%) | 19 (5.5%) |
| Australia | 94 (7.1%) | 54 (7.3%) | 34 (9.9%) |
| North America | 541 (41%) | 284 (38%) | 151 (44%) |
| Other | 25 (1.9%) | 9 (1.2%) | 0 (0%) |
| **Race** |  |  |  |
| White | 898 (68%) | 514 (69%) | 270 (78%) |
| Black | 283 (22%) | 148 (20%) | 35 (10%) |
| Other | 134 (10%) | 81 (11%) | 40 (12%) |
| **Marital Status** |  |  |  |
| Married or Living With Partner | 976 (86%) | 572 (84%) | 273 (85%) |
| Not Married | 157 (14%) | 109 (16%) | 49 (15%) |
| Missing (n) | 182 | 63 | 24 |
| **Employment Status** |  |  |  |
| Working | 310 (28%) | 188 (28%) | 92 (29%) |
| Unemployed or Other | 109 (9.7%) | 59 (8.7%) | 16 (5.0%) |
| Retired | 701 (63%) | 432 (64%) | 211 (66%) |
| Missing (n) | 195 | 65 | 27 |
| **Education Level** |  |  |  |
| Higher Education | 430 (41%) | 280 (44%) | 133 (43%) |
| Less than higher education | 619 (59%) | 361 (56%) | 179 (57%) |
| Missing (n) | 266 | 103 | 34 |
| **EORTC QLQ-C30 Summary Score** | 87.1 (17.7) | 88.0 (16.0) | 89.8 (14.6) |
| Missing (n) | 55 | 24 | 10 |
| **PSA (ng/mL)** | 6.18 (32.78) | 6.12 (24.22) | 3.97 (18.18) |
| Missing (n) | 312 | 133 | 54 |
| **ECOG Performance Status** |  |  |  |
| 0 | 539 (54%) | 361 (56%) | 205 (70%) |
| 1 | 319 (32%) | 221 (35%) | 78 (27%) |
| 2 | 93 (9.4%) | 31 (4.9%) | 7 (2.4%) |
| 3 | 32 (3.2%) | 22 (3.4%) | 2 (0.7%) |
| 4 | 8 (0.8%) | 4 (0.6%) | 1 (0.3%) |
| Missing (n) | 324 | 105 | 53 |
| **Disease State** |  |  |  |
| mHSPC | 960 (73%) | 567 (76%) | 266 (77%) |
| CRPC | 352 (27%) | 176 (24%) | 80 (23%) |
| Missing (n) | 3 | 1 | 0 |
| **First On-Study Treatment Regimen** |  |  |  |
| ADT + ARPI | 668 (58%) | 413 (60%) | 202 (64%) |
| ADT + ARPI + chemo | 89 (7.8%) | 54 (7.9%) | 13 (4.1%) |
| ADT + chemo | 139 (12%) | 66 (9.6%) | 46 (14%) |
| ADT monotherapy | 250 (22%) | 152 (22%) | 57 (18%) |
| Missing (n) | 169 | 59 | 28 |
| **Time on Study (Months)** | 15 (30) | 27 (18) | 39 (12) |

Continuous variables are presented as median (interquartile range), and categorical variables as counts (percentages). Abbreviations: BMI: Body Mass Index; EORTC QLQ-C30: European Organisation for Research and Treatment of Cancer Quality of Life Questionnaire-Core 30; PSA: Prostate-Specific Antigen; ECOG: Eastern Cooperative Oncology Group; ADT: Androgen Deprivation Therapy; ARPI: Androgen Receptor Pathway Inhibitor; Chemo: Chemotherapy; mHSPC: Metastatic Hormone-Sensitive Prostate Cancer; CRPC: Castration-Resistant Prostate Cancer.

Supplemental Table S3. Baseline characteristics of patients with advanced prostate cancer in IRONMAN Registry, stratified by Racial Subgroups

| **Characteristic** | **White  N = 1,729** | **Black  N = 473** | **Other  N = 283** |
| --- | --- | --- | --- |
| **Age (years)** | 71.0 (11.0) | 67.0 (11.0) | 70.0 (12.0) |
| **BMI** | 27.3 (5.4) | 25.0 (5.9) | 27.4 (4.5) |
| Missing (n) | 518 | 197 | 145 |
| **Geographic Region** |  |  |  |
| Europe | 750 (43%) | 6 (1.3%) | 177 (63%) |
| Africa | 4 (0.2%) | 292 (62%) | 11 (3.9%) |
| Australia | 169 (9.8%) | 0 (0%) | 18 (6.4%) |
| North America | 794 (46%) | 155 (33%) | 74 (26%) |
| Other | 12 (0.7%) | 20 (4.2%) | 3 (1.1%) |
| **Marital Status** |  |  |  |
| Married or Living With Partner | 1,313 (84%) | 373 (88%) | 149 (86%) |
| Not Married | 244 (16%) | 49 (12%) | 24 (14%) |
| Missing (n) | 172 | 51 | 110 |
| **Employment Status** |  |  |  |
| Working | 453 (29%) | 99 (24%) | 44 (25%) |
| Unemployed or Other | 79 (5.1%) | 95 (23%) | 10 (5.8%) |
| Retired | 1,017 (66%) | 218 (53%) | 119 (69%) |
| Missing (n) | 180 | 61 | 110 |
| **Education Level** |  |  |  |
| Higher Education | 681 (47%) | 130 (32%) | 42 (26%) |
| Less than higher education | 774 (53%) | 273 (68%) | 118 (74%) |
| Missing (n) | 274 | 70 | 123 |
| **EORTC QLQ-C30 Summary Score** | 88.6 (15.2) | 84.1 (23.2) | 87.7 (15.9) |
| Missing (n) | 39 | 36 | 15 |
| **PSA (ng/mL)** | 3.90 (17.33) | 33.00 (95.33) | 4.44 (19.36) |
| Missing (n) | 302 | 170 | 55 |
| **ECOG Performance Status** |  |  |  |
| 0 | 883 (66%) | 92 (24%) | 130 (67%) |
| 1 | 421 (31%) | 138 (36%) | 57 (29%) |
| 2 | 28 (2.1%) | 97 (25%) | 6 (3.1%) |
| 3 | 7 (0.5%) | 48 (12%) | 1 (0.5%) |
| 4 | 1 (<0.1%) | 12 (3.1%) | 0 (0%) |
| Missing (n) | 389 | 86 | 89 |
| **Disease State** |  |  |  |
| mHSPC | 1,291 (75%) | 356 (75%) | 210 (75%) |
| CRPC | 436 (25%) | 117 (25%) | 71 (25%) |
| Missing (n) | 2 | 0 | 2 |
| **First On-Study Treatment Regimen** |  |  |  |
| ADT + ARPI | 1,011 (64%) | 151 (38%) | 157 (62%) |
| ADT + ARPI + chemo | 133 (8.4%) | 12 (3.1%) | 19 (7.5%) |
| ADT + chemo | 190 (12%) | 29 (7.4%) | 41 (16%) |
| ADT monotherapy | 240 (15%) | 201 (51%) | 35 (14%) |
| Missing (n) | 155 | 80 | 31 |
| **Time on Study (Months)** | 30 (27) | 18 (15) | 27 (21) |

Continuous variables are presented as median (interquartile range), and categorical variables as counts (percentages). Abbreviations: BMI: Body Mass Index; EORTC QLQ-C30: European Organisation for Research and Treatment of Cancer Quality of Life Questionnaire-Core 30; PSA: Prostate-Specific Antigen; ECOG: Eastern Cooperative Oncology Group; ADT: Androgen Deprivation Therapy; ARPI: Androgen Receptor Pathway Inhibitor; Chemo: Chemotherapy; mHSPC: Metastatic Hormone-Sensitive Prostate Cancer; CRPC: Castration-Resistant Prostate Cancer.

Supplemental Table S4. Baseline characteristics of patients with advanced prostate cancer in IRONMAN Registry, stratified by Geographic Region.

| **Characteristic** | **Europe  N = 933^1^** | **Africa  N = 307^1^** | **Australia  N = 187^1^** | **North America  N = 1,023^1^** | **Other  N = 35^1^** |  |
| --- | --- | --- | --- | --- | --- | --- |
| **Age (years)** | 71.0 (11.0) | 67.0 (10.0) | 70.0 (12.0) | 70.0 (11.0) | 70.0 (11.5) |  |
| **BMI** | 26.9 (4.6) | 23.9 (6.0) | 27.8 (6.4) | 27.8 (5.9) | 25.8 (6.4) |  |
| Missing (n) | 304 | 129 | 70 | 341 | 16 |  |
| **Race** |  |  |  |  |  |  |
| White | 750 (80%) | 4 (1.3%) | 169 (90%) | 794 (78%) | 12 (34%) |  |
| Black | 6 (0.6%) | 292 (95%) | 0 (0%) | 155 (15%) | 20 (57%) |  |
| Other | 177 (19%) | 11 (3.6%) | 18 (9.6%) | 74 (7.2%) | 3 (8.6%) |  |
| **Marital Status** |  |  |  |  |  |  |
| Married or Living With Partner | 678 (84%) | 270 (97%) | 137 (81%) | 728 (83%) | 22 (79%) |  |
| Not Married | 128 (16%) | 7 (2.5%) | 32 (19%) | 144 (17%) | 6 (21%) |  |
| Missing (n) | 127 | 30 | 18 | 151 | 7 |  |
| **Employment Status** |  |  |  |  |  |  |
| Working | 181 (23%) | 53 (20%) | 60 (36%) | 293 (34%) | 9 (32%) |  |
| Unemployed or Other | 31 (3.9%) | 70 (26%) | 15 (8.9%) | 65 (7.5%) | 3 (11%) |  |
| Retired | 590 (74%) | 144 (54%) | 93 (55%) | 511 (59%) | 16 (57%) |  |
| Missing (n) | 131 | 40 | 19 | 154 | 7 |  |
| **Education Level** |  |  |  |  |  |  |
| Higher Education | 276 (36%) | 78 (30%) | 61 (38%) | 434 (55%) | 4 (14%) |  |
| Less than higher education | 497 (64%) | 186 (70%) | 101 (62%) | 357 (45%) | 24 (86%) |  |
| Missing (n) | 160 | 43 | 25 | 232 | 7 |  |
| **EORTC QLQ-C30 Summary Score** | 89.4 (14.1) | 81.6 (24.7) | 84.4 (15.7) | 88.2 (15.9) | 88.2 (11.7) |  |
| Missing (n) | 29 | 35 | 6 | 19 | 1 |  |
| **PSA (ng/mL)** | 3.50 (15.22) | 69.12 (87.00) | 5.30 (16.25) | 5.49 (22.84) | 6.99 (13.71) |  |
| Missing (n) | 132 | 129 | 43 | 209 | 14 |  |
| **ECOG Performance Status** |  |  |  |  |  |  |
| 0 | 522 (72%) | 27 (11%) | 82 (62%) | 463 (59%) | 11 (58%) |  |
| 1 | 193 (27%) | 81 (32%) | 45 (34%) | 290 (37%) | 7 (37%) |  |
| 2 | 7 (1.0%) | 88 (35%) | 6 (4.5%) | 30 (3.8%) | 0 (0%) |  |
| 3 | 3 (0.4%) | 47 (18%) | 0 (0%) | 5 (0.6%) | 1 (5.3%) |  |
| 4 | 0 (0%) | 12 (4.7%) | 0 (0%) | 1 (0.1%) | 0 (0%) |  |
| Missing (n) | 208 | 52 | 54 | 234 | 16 |  |
| **Disease State** |  |  |  |  |  |  |
| mHSPC | 696 (75%) | 231 (75%) | 121 (66%) | 791 (77%) | 18 (51%) |  |
| CRPC | 236 (25%) | 76 (25%) | 63 (34%) | 232 (23%) | 17 (49%) |  |
| Missing (n) | 1 | 0 | 3 | 0 | 0 |  |
| **First On-Study Treatment Regimen** |  |  |  |  |  |  |
| ADT + ARPI | 605 (69%) | 62 (26%) | 61 (42%) | 585 (62%) | 6 (30%) |  |
| ADT + ARPI + chemo | 80 (9.2%) | 1 (0.4%) | 4 (2.7%) | 79 (8.4%) | 0 (0%) |  |
| ADT + chemo | 111 (13%) | 11 (4.6%) | 38 (26%) | 98 (10%) | 2 (10%) |  |
| ADT monotherapy | 78 (8.9%) | 166 (69%) | 43 (29%) | 177 (19%) | 12 (60%) |  |
| Missing (n) | 59 | 67 | 41 | 84 | 15 |  |
| **Time on Study (Months)** | 27 (24) | 15 (12) | 30 (32) | 30 (30) | 21 (26) |  |

Continuous variables are presented as median (interquartile range), and categorical variables as counts (percentages). Abbreviations: BMI: Body Mass Index; EORTC QLQ-C30: European Organisation for Research and Treatment of Cancer Quality of Life Questionnaire-Core 30; PSA: Prostate-Specific Antigen; ECOG: Eastern Cooperative Oncology Group; ADT: Androgen Deprivation Therapy; ARPI: Androgen Receptor Pathway Inhibitor; Chemo: Chemotherapy; mHSPC: Metastatic Hormone-Sensitive Prostate Cancer; CRPC: Castration-Resistant Prostate Cancer.

Supplemental Table S5. Sensitivity Analysis: Exclude Black participants from Africa in analysis for race.

|  |  | Aerobic Physical Activity Guidelines | | Resistance Training Guidelines | |
| --- | --- | --- | --- | --- | --- |
| Subgroup Type | Subgroup | cOR (95% CI) | aOR (95% CI) | cOR (95% CI) | aOR (95% CI) |
| Race (ref = White) | Black | 0.29 (0.18 - 0.46) | 0.3 (0.19 - 0.47) | 0.99 (0.29 - 3.32) | 1.06 (0.31 - 3.66) |
|  | Other | 0.65 (0.47 - 0.9) | 0.67 (0.48 - 0.93) | 0.62 (0.22 - 1.73) | 0.62 (0.21 - 1.8) |

Odds ratios (OR) and 95% confidence intervals (CI) are shown for subgroup comparisons. An OR > 1 indicates higher odds of meeting guidelines compared to the reference subgroup across timepoints. Crude odds ratios (cOR) were adjusted for timepoint (fixed effects) and includes a random intercept for the participant to account for within-subject variation. Adjusted odds ratios (aOR) were further adjusted for age, education, ECOG status, and first on-study treatment. Statistically significant (p < 0.05) odds ratios are highlighted with **bold** font. Abbreviations: ref = reference.

Supplemental Table S6. Sensitivity Analysis: exclude participants with only 1 valid timepoint

|  |  | Aerobic Physical Activity Guidelines | | Resistance Training Guidelines | |
| --- | --- | --- | --- | --- | --- |
| Subgroup Type | Subgroup | cOR (95% CI) | aOR (95% CI) | cOR (95% CI) | aOR (95% CI) |
| Race (ref = White) | Black | 0.20 (0.11 - 0.38) | 0.22 (0.13 - 0.37) | 0.25 (0.08 - 0.78) | 0.36 (0.03 - 4.77) |
|  | Other | 0.63 (0.4 - 1.01) | 0.65 (0.42 - 1) | 0.56 (0.19 - 1.62) | 0.50 (0.06 - 4.2) |
| Geographic Region (ref = Europe) | Australia | 0.97 (0.39 - 2.42) | 1.16 (0.49 - 2.75) | 1.84 (0.5 - 6.75) | 4.68 (1.25 - 17.47) |
|  | North America | 1.11 (0.62 - 2) | 1.13 (0.65 - 1.95) | 1.56 (0.74 - 3.29) | 2.06 (1 - 4.25) |
|  | Africa | 0.06 (0.02 - 0.17) | 0.13 (0.04 - 0.36) | 0.030 (0 - 1.07) |  |

Odds ratios (OR) and 95% confidence intervals (CI) are shown for subgroup comparisons. An OR > 1 indicates higher odds of meeting guidelines compared to the reference subgroup across timepoints. Crude odds ratios (cOR) were adjusted for timepoint (fixed effects) and includes a random intercept for the participant to account for within-subject variation. Adjusted odds ratios (aOR) were further adjusted for age, education, ECOG status, and first on-study treatment. Statistically significant (p < 0.05) odds ratios are highlighted with **bold** font. Empty cells are due to convergence issues. Abbreviations: ref = reference.

Supplemental Table S7 Sensitivity Analysis: exclude observations at Month 30.

|  |  | Aerobic Physical Activity Guidelines | | Resistance Training Guidelines | |
| --- | --- | --- | --- | --- | --- |
| Subgroup Type | Subgroup | cOR (95% CI) | aOR (95% CI) | cOR (95% CI) | aOR (95% CI) |
| Race (ref = White) | Black | 0.22 (0.15 - 0.34) | 0.25 (0.16 - 0.38) | 0.26 (0.26 - 0.26) | 0.37 (0.09 - 1.45) |
|  | Other | 0.76 (0.55 - 1.04) | 0.77 (0.55 - 1.07) | 0.61 (0.2 - 1.89) | 0.64 (0.19 - 2.2) |
| Geographic Region (ref = Europe) | Australia | 1.22 (0.58 - 2.57) | 1.41 (0.68 - 2.91) | 1.65 (0.55 - 4.99) | 2.30 (0.02 - 216.01) |
|  | North America | 0.87 (0.55 - 1.39) | 0.91 (0.58 - 1.43) | 1.30 (0.77 - 2.18) | 1.53 (0.1 - 24.05) |
|  | Africa | 0.1 (0.04 - 0.25) | 0.19 (0.08 - 0.45) | 0.08 (0.08 - 0.08) |  |

Odds ratios (OR) and 95% confidence intervals (CI) are shown for subgroup comparisons. An OR > 1 indicates higher odds of meeting guidelines compared to the reference subgroup across timepoints. Crude odds ratios (cOR) were adjusted for timepoint (fixed effects) and includes a random intercept for the participant to account for within-subject variation. Adjusted odds ratios (aOR) were further adjusted for age, education, ECOG status, and first on-study treatment. Statistically significant (p < 0.05) odds ratios are highlighted with **bold** font. Empty cells are due to convergence issues. Abbreviations: ref = reference.

Supplemental Table S8. Findings from mixed effects logistic regression for odds of meeting physical activity guidelines across timepoints stratified by disease state,

|  |  |  | **Aerobic Physical Activity Guidelines** | | **Resistance Training Guidelines** | |
| --- | --- | --- | --- | --- | --- | --- |
| **Disease State** | **Subgroup Type** | **Subgroup** | **cOR (95% CI)** | **aOR (95% CI)** | **cOR (95% CI)** | **aOR (95% CI)** |
| mHSPC | Race (ref = White) | Black | **0.19 (0.11 - 0.32)** | **0.21 (0.12 - 0.34)** | **0.26 (0.08 - 0.86)** | 0.36 (0.1 - 1.29) |
|  |  | Other | **0.57 (0.38 - 0.83)** | **0.62 (0.42 - 0.91)** | 0.56 (0.17 - 1.78) | 0.56 (0.17 - 1.85) |
|  | Geographic Region (ref = Europe) | Australia | 1.54 (0.64 - 3.7) | 1.64 (0.67 - 3.99) | 1.45 (0.38 - 5.57) | 1.44 (0.37 - 5.65) |
|  |  | North America | 0.91 (0.54 - 1.54) | 0.95 (0.56 - 1.62) | 1.43 (0.68 - 2.99) | 1.41 (0.65 - 3.05) |
|  |  | Africa | **0.06 (0.02 - 0.16)** | **0.12 (0.04 - 0.34)** | 0.05 (0 - 1.27) | 0.06 (0 - 1.73) |
| CRPC | Race (ref = White) | Black | **0.29 (0.16 - 0.53)** | **0.36 (0.19 - 0.67)** | 0.98 (0.05 - 20.37) | 1.17 (0.06 - 24.85) |
|  |  | Other | 0.99 (0.56 - 1.76) | 0.89 (0.48 - 1.65) | 0.51 (0.03 - 8.36) | 0.57 (0.03 - 9.86) |
|  | Geographic Region (ref = Europe) | Australia | 0.67 (0.34 - 1.3) | 0.70 (0.35 - 1.38) | 1.98 (0.23 - 17.47) | 2.14 (0.23 - 19.89) |
|  |  | North America | **0.56 (0.36 - 0.88)** | **0.48 (0.3 - 0.77)** | 0.85 (0.17 - 4.3) | 0.76 (0.14 - 3.98) |
|  |  | Africa | **0.23 (0.11 - 0.48)** | **0.30 (0.13 - 0.66)** |  |  |

Odds ratios (OR) and 95% confidence intervals (CI) are shown for subgroup comparisons. An OR > 1 indicates higher odds of meeting guidelines compared to the reference subgroup across timepoints. Crude odds ratios (cOR) were adjusted for timepoint (fixed effects) and includes a random intercept for the participant to account for within-subject variation. Adjusted odds ratios (aOR) were further adjusted for age, education, ECOG status, and first on-study treatment. Statistically significant (p < 0.05) odds ratios are highlighted with **bold** font. Empty cells are due to convergence issues. Abbreviations: ref = reference; mHSPC: metastatic hormone-sensitive prostate cancer; ADT: CRPC: castration-resistant prostate cancer.
